# Building back better? Taking stock of the post-earthquake mental health and psychosocial response in Nepal

**DOI:** 10.1186/s13033-018-0221-3

**Published:** 2018-08-01

**Authors:** Liana E. Chase, Kedar Marahatta, Kripa Sidgel, Sujan Shrestha, Kamal Gautam, Nagendra P. Luitel, Bhogendra Raj Dotel, Reuben Samuel

**Affiliations:** 1 Department of Anthropology, SOAS University of London, 10 Thornhaugh Street, Russell Square, London, WC1H 0XG UK; 2 WHO Health Emergencies Programme, WHO Country Office for Nepal, UN House, Lalitpur, Nepal; 3 Psychbigyaan Network Nepal, Chakrapath-3, Kathmandu, Nepal; 4 Transcultural Psychosocial Organization Nepal, Baluwatar, Kathmandu, Nepal; 5 Primary Health Care Revitalization Division, Department of Health Services, Ministry of Health and Population, Kathmandu, Nepal; 6 WHO Health Emergencies Programme, WHO Country Office for Nepal, UN House, Lalitpur, Nepal

**Keywords:** Disaster, Mental health systems, Nepal, Building back better, Sustainability, Mental health and psychosocial support, Intervention, Earthquake

## Abstract

**Background:**

The World Health Organization’s ‘building back better’ approach advocates capitalizing on the resources and political will elicited by disasters to strengthen national mental health systems. This study explores the contributions of the response to the 2015 earthquake in Nepal to sustainable mental health system reform.

**Methods:**

We systematically reviewed grey literature on the mental health and psychosocial response to the earthquake obtained through online information-sharing platforms and response coordinators (168 documents) to extract data on response stakeholders and activities. More detailed data on activity outcomes were solicited from organizations identified as most active in the response. To triangulate and extend findings, we held a focus group discussion with key governmental and non-governmental stakeholders in mental health system development in Nepal (n = 10). Discussion content was recorded, transcribed, and subjected to thematic analysis.

**Results:**

While detailed documentation of response activities was limited, available data combined with stakeholders’ accounts suggest that the post-earthquake response accelerated progress towards national mental health system building in the areas of governance, financing, human resources, information and research, service delivery, and medications. Key achievements in the post-earthquake context include training of primary health care service providers in affected districts using mhGAP and training of new psychosocial workers; appointment of mental health focal points in the government and World Health Organization Country Office; the addition of new psychotropic drugs to the government’s free drugs list; development of a community mental health care package and training curricula for different cadres of health workers; and the revision of mental health plans, policy, and financing mechanisms. Concerns remain that government ownership and financing will be insufficient to sustain services in affected districts and scale them up to non-affected districts.

**Conclusions:**

Building back better has been achieved to varying extents in different districts and at different levels of the mental health system. Non-governmental organizations and the World Health Organization Country Office must continue to support the government to ensure that recent advances maximally contribute to realising the vision of a national mental health care system in Nepal.

## Background

Since the late 1980s, humanitarian responses to disasters have generally incorporated a mental health and psychosocial support (MHPSS) component [1]. In contexts where existing mental health services are limited, such responses have often involved an influx of professionals and expertise from other countries and the establishment of vertical trauma-focused services [2–5]. Scholars have widely interrogated the clinical effectiveness, contextual appropriateness, and sustainability of this model, highlighting its potential to undermine existing community supports, coping strategies, and health services [6–9].

Over the past decade, a new discourse on emergency MHPSS response has taken shape which emphasises the potential for ‘building back better’, or channelling the resources and political will elicited by disasters towards national mental health system building agendas [10, 11]. Evidence from a variety of contexts supports the World Health Organization’s (WHO) assertion that disasters offer crucial opportunities for sustainable mental health system reform. Following the tsunami in Indonesia, for example, health officials decided to implement community-based mental health services in the affected province of Aceh when NGOs providing emergency mental health services withdrew [10]. The province has now become a role model for the rest of the country [11]. In post-tsunami Sri Lanka, both the head of state and key actors in the Ministry of Health, encouraged by international media, recognized an imperative to respond to the mental health needs of survivors; their efforts led to the passing of a new national mental health policy calling for a community-based, comprehensive and decentralized care system [10]. Following conflict in Burundi and Afghanistan, NGOs providing most of the country’s mental health care were able to successfully transfer capacity and responsibility to the government [10]. By contrast, after the 2010 earthquake in Haiti, the government’s lack of interest and engagement in mental health led practitioners to question whether it was wise to invest in community mental health services and the training of primary care providers [12].

Efforts have now been made to document lessons learnt from previous disasters and best practices for effectively transitioning from emergency response to sustainable mental health system development. Leading guidelines on MHPSS in emergency settings now call for capacity-building, strengthening of community supports, and integration within existing public services [13, 14]. The WHO advocates planning for sustainability and holistic mental health system strengthening from the outset, respecting the central role of the government and local mental health professionals, training and reorganizing health workers, and reviewing and revising national mental health policy and plans [10]. The MHPSS working group of the Harvard Humanitarian Initiative further underscores the importance of recognizing and leveraging existing knowledge and capacity and integrating post-disaster programs into existing services [15]. There is a lack of evidence on how fully and successfully these guidelines have been applied across diverse disaster-affected settings since their publication.

### Study context

Nepal is a geographically and ethnically diverse country with the third lowest human development ranking in South Asia [16]. Formal mental health services and specialized human resources are extremely limited [17] and non-governmental organizations (NGOs) have historically played a leading role in MHPSS training and intervention [18]. The United Mission to Nepal introduced the first community mental health services in the 1980s [19, 20]. In the l990s and early 2000s, NGOs such as the Centre for Victims of Torture-Nepal, the Centre for Mental Health and Counselling-Nepal, and the Transcultural Psychosocial Organisation Nepal began providing mental health and psychosocial care to victims of the ongoing civil conflict and Bhutanese refugee crisis [21–23]. A key output of their efforts was the development of a culturally adapted model of psychosocial care and associated training curricula [21, 24, 25]. In this model, ‘psychosocial counsellors’ (typically with 6 months of training) provide clients basic emotional support and assistance with problem solving while ‘community psychosocial workers’ with a few days to a few weeks of training identify and refer cases to counsellors and may themselves provide some basic emotional support [21]. While NGOs in Nepal adopted many best practices from the outset—including cultural adaptation and community-based service delivery—their contributions were often piecemeal and unsustainable due to lack of government involvement and oversight [18].

Over the past decade important strides have been made toward developing a national community mental health system through the concerted efforts of NGOs, the WHO Country Office, the government, and international global mental health projects: an existing model of community mental health care in Nepal was further refined, piloted, and evaluated in the public health system [26–28] and the government identified mental health as a priority area in its multi-sectorial action plan for the prevention and control of non-communicable diseases (NCDs) (2014–2020) [29]. However, implementation of the action plan and scale up of the community mental health model were stymied by political instability and governance issues including high staff turnover, lack of a focal point for mental health, and inadequate budget and spending capacity.

In spring 2015, Nepal was struck by a high-magnitude earthquake followed by a major aftershock. Fourteen of Nepal’s 77 districts were highly affected and nearly 9000 people lost their lives [30, 31]. Needs assessments after the earthquake revealed high rates of depression, anxiety, suicidal ideation and hazardous alcohol use relative to other disaster-affected populations and WHO estimates; however, relatively low rates of corresponding functional impairment and PTSD suggest that many participants were experiencing psychological distress, rather than disorder [32, 33]. The months that followed witnessed an ‘unprecedented’ investment in mental health by governmental and non-governmental stakeholders [34]. A desk review of information relevant to MHPSS in Nepal was prepared [35]. A cluster system was established including both psychosocial and mental health subclusters and mechanisms were put in place to coordinate and share information related to the MHPSS response, including submission of 5W (Who, What, Where, When, for Whom) forms detailing response activities to the subcluster chairs; regular subcluster meetings and circulation of meeting minutes; and sections of the online platforms MHPSS.net and humanitarianresponse.info.

Sherchan and colleagues [36] have published a detailed account of the coordination and leadership of the MHPSS response to the earthquakes in which they reflect on successes and challenges through the lens of their first-hand experience as coordinators. The present study complements this analysis by considering longer-term implications of the disaster and associated response and recovery activities for mental health system building. First, we reviewed documents from multiple grey literature sources to extract data on post-earthquake MHPSS response activities. Then, we held a focus group discussion with key governmental and non-governmental actors in mental health system development. By triangulating and extending data on response activity outcomes with primary qualitative data from key stakeholders, this article aims to take an empirical look at the extent to which building back better has been achieved in Nepal.

## Methods

### Grey literature review

In light of the aims of our review (extracting data on MHPSS response activity outcomes rather than synthesizing scholarly knowledge), and after extensive preliminary searches in scholarly databases yielded no relevant results, we opted for a targeted review of grey literature in lieu of a full systematic review methodology. Through discussion with response coordinators, two databases were identified that had been widely used to share information about post-earthquake MHPSS response activities: MHPSS.net and humanitarianresponse.info. Full texts of all documents shared in Nepal earthquake groups/sections of both online platforms were downloaded in June 2017 and duplicates were removed manually. Then, mental health and psychosocial subcluster chairs were contacted to obtain any additional available documentation of post-earthquake MHPSS response activities.

#### Data analysis

Two research assistants and the lead investigator divided all documents obtained for full-text review. Basic characteristics of all documents (e.g., language) were entered into a shared spreadsheet. When any document contained information on a MHPSS disaster response activity, this information was extracted and entered into a second shared spreadsheet. For the purposes of this review, a MHPSS disaster response activity was defined as any effort to provide or improve access to mental health and/or psychosocial care in earthquake-affected populations. Activity data extracted included both descriptive details (e.g., funding organization, start and end dates) and activity outcomes (e.g., primary care workers trained on mhGAP, psychosocial counselling provided). Information was disaggregated both project-wise and district-wise whenever possible. The lead investigator reviewed a subset of entries made by research assistants to ensure accuracy and consistency. After the limitations of available documentation were noted (see “Results” section), email requests for more accurate and detailed response activity data were sent to eight organizations highly active in the response. When these yielded minimal response, more targeted requests were made (in person or over the phone when necessary) to obtain data from the three mental health organizations identified through the document review as most active in the response. Data obtained from all three organizations covered all post-earthquake MHPSS response activities implemented between the time of the earthquakes and the end of 2016. One organization provided an internal report on its post-earthquake activities, from which data was extracted following the procedure described above. Two organizations extracted data internally and provided final figures for the total number of beneficiaries reached for each type of post-earthquake activity they had conducted. Data for each activity type from the three organizations were then summed.

### Focus group discussion

In order to triangulate and extend findings of the grey literature review, we organized a 2-h focus group discussion with key stakeholders in Nepal’s mental health sector in August 2017. An invitation list was prepared by the first and second authors based on the review findings and their knowledge of Nepal’s mental health sector and included 12 individuals and institutions that have been active in mental health system building in both pre- and post-earthquake contexts. Two invitees could not attend due to conflicts. Participants who attended (n = 10) were mostly in senior management positions in their organizations and included one clinical psychologist, five psychiatrists, one public health officer, two mental health activists/service users, and one non-clinician executive director of an organization. Organizations represented included the WHO Country Office, two government hospitals, a service users’ organization, two NGOs (both among the three providing data on activities), and the Government of Nepal Ministry of Health (MOH), Department of Health Services.

An interview guide was developed based on the WHO’s health system building blocks: governance, financing, human resources, information and research, service delivery, and medicines. For each of these areas, participants were prompted to discuss pre- and post-earthquake developments and the contribution, if any, of MHPSS earthquake response and recovery activities to recent advances. Additional prompts addressed stakeholders’ perceptions of the sustainability of progress made.

#### Data analysis

Interview data were audio recorded, transcribed, and subject to thematic content analysis following Green and Thorogood [37]. Themes were derived deductively based on the interview guide topics (e.g., ‘sustainability of recent progress’, ‘governance’, ‘financing’, etc.). Both the lead investigator and a research assistant completed a full coding of the transcript. Discrepancies in coding were identified through manual comparison and resolved through discussion.

## Results

### Outcomes of post-earthquake MHPSS response activities

We identified and reviewed 168 documents, of which 47 reported on MHPSS response activities. With one exception, all documents provided by response coordinators had already been identified through the databases. Characteristics and sources of documents reviewed are depicted in Table 1. Review of these documents identified 56 stakeholders in the post-disaster MHPSS response (Table 2) and a range of activity outcomes spanning direct service provision, capacity building, and psychoeducation. However, information on activity length, geographic coverage, and beneficiaries was not widely reported. There was also ambiguity over activity content; for example, some organizations used ‘psychosocial support’ to describe non-specific, non-clinical activities such as good parenting sessions while others used the term in reference to structured clinical activities. It was furthermore often impossible to discern when an activity had been reported on by both funding and implementing organisations.

**Table 1:** Overview of documents reviewed

Document characteristics	Number of documents^a^
*Material source*	
MHPSS.net	141
Humanitarianresponse.info	26
Response coordinator	1
*Material type*	
Situation report	42
MHPSS intervention tool	16
Cluster/working group meeting minutes and updates	24
Resource list	6
Report on response activities	10
4W or 5W mastersheet^b^	3
Screening tool	4
Research report/article	24
Disaster response guidelines/reference document (not specific to MHPSS)	16
MHPSS response guidelines/reference document	21
Other	2
*Language*	
Nepali	7
English	155
Nepali and English	5
Other	1
*Relevance*	
Has been adapted for or is specific to use in Nepali context	124
Contains information about response activities	47

^a^After manually excluding duplicates

^b^Spreadsheet integrating information from all 5W (4W in earlier versions) forms submitted to subcluster coordinators

**Table 2:** Stakeholders in post-earthquake MHPSS response

Stakeholder type	Number identified
National NGO	22
International NGO	20
UN body	7
Hospital	4
Independent practitioner	1
Other	2
Total	56

NB: A ‘stakeholder’ was defined as any organization that reported funding and/or implementing one or more post-earthquake MHPSS response activities

In order to gain a more accurate and fine-grained picture of post-earthquake MHPSS response activities and outcomes, we solicited data from the three mental health organizations identified as most active in the response: Transcultural Psychosocial Organization Nepal, Centre for Victims of Torture-Nepal, and the Centre for Mental Health and Counselling-Nepal; this data is summarized in Table 3. In addition to outcomes that could be quantified, these organisations reported a range of non-specific psychoeducation activities including dissemination of ‘Information, Education, and Communication’ materials on mental health, community theatre performances, radio programs, anti-stigma campaigns, and the construction of billboards on mhGAP priority disorders.

**Table 3:** MHPSS response activity outcomes of three most active mental health organizations

Activity type	Activity	Beneficiaries
Direct service provision	Psychological first aid (PFA)	66,175
	Psychosocial Counselling or Support^a^	69,987
	Psychiatric treatment	363
	Mental health services from trained primary health provider^b^	3655
	Total	140,180
Capacity building	Training on providing psychological first aid	2098
	New community psychosocial workers^c^	741
	New psychosocial counsellors^d^	56
	Supplementary Training for Psychosocial Counsellors	66
	Primary Health Care Providers (Prescribers) Trained^e^	642
	Primary Health Care Providers (Non-Prescribers) Trained^e^	348
	Female Community Health Volunteers and Auxiliary Nurse Midwives^f^	2285
	Total	6236
Awareness raising	Trainings/orientations for frontline workers and community leaders^g^	7018
	Psychoeducation/orientation for general community members^h^	131,701
	Total	138,719
All activities	Total	285,135

^a^Includes individual, family, and group counselling and psychosocial support provided by psychosocial counsellors, community psychosocial workers, and other facilitators who had received training comparable to or longer than that of community psychosocial workers

^b^Refers to number of people who received consultation, assessment, and management as needed for mental health problems from primary care workers trained in mental health (see ‘Capacity building’); data reported by only one organization for four districts in which it was working

^c^Trainings ranged from 3 days (with follow-up module) to 20 days

^d^This is generally a 6-month course, with some variation in ratio of practical to theoretical content

^e^Training was based on mhGAP Humanitarian Intervention Guide (HIG) with some additional modules on psychosocial care

^f^Trainings of several days focused on identification and referral and basic psychosocial support skills

^g^Includes trainings and orientations targeting teachers, traditional healers, frontline workers, police, local politicians, social mobilisers, protection actors, and various other health workers and community leaders

^h^Includes participants at community meetings and orientations and individuals who were directly provided psychoeducational paper materials

### Stakeholders’ perspectives

#### Accelerated mental health system building

There was strong consensus among stakeholders that the earthquake had accelerated processes of mental health system building that were already underway before 2015. Participants described a number of important developments in the mental health sector prior to the earthquake that were crucial to ensuring an effective MHPSS post-earthquake response and subsequent developments. However, they also emphasized that pre-earthquake progress had been piecemeal and largely unsustainable due to lack of investment and coordination by the government. One participant summarized, ‘…all mental health professionals felt we have to do this, but there was no focal point, no financing, there was no support to do this work, from many angles it wasn’t there’.

The earthquake, and associated response and recovery activities, were credited with increasing the pace of progress towards the vision and goals established through these earlier efforts. One stakeholder estimated, ‘The earthquake accelerated this more than 50%’. This was mainly ascribed to indirect effects of the earthquake—specifically, to increased awareness of the importance of mental health, especially among key funders and decision-makers. As one participant explained, ‘In the pre-earthquake period the role of psychiatrists and psychologists and the importance of mental health was very low priority—the government didn’t care and the public didn’t care. Now…there has been a shift I feel’. In addition, stakeholders mentioned several direct outputs of response activities, including the training of primary care providers and psychosocial counsellors in affected districts. A summary of pre- and post-earthquake developments reported by stakeholders is provided in Table 4 along with quotes reflecting the specific contributions of the earthquake and associated response and recovery activities.

**Table 4:** Pre- and post-earthquake developments in MHPSS system building

Health system building block	Pre-earthquake developments	Post-earthquake developments	Contribution of the post-earthquake MHSS response: representative quotes
Governance	• National mental health policy endorsed (1997) • Mental health legislation drafted but not passed • Multi-Sectoral Action Plan for the Prevention and Control of NCDs (2014–2020) including mental health endorsed but poorly implemented	• Revised mental health policy drafted by MOH; endorsement by cabinet pending • Mental health legislation being drafted through a collaborative effort among stakeholders • Government focal point for mental health assigned: Primary Health Care Revitalisation Division (PHCRD) • Mental health focal point continually available in WHO Country Office • Community Mental Health Care Package Nepal 2074 detailing minimum standards for mental health in primary care prepared^a^	*‘If the earthquake hadn’t happened, the government would not have brought rules. All NGOs, INGOs would work on their own behalf, in different sites, which is duplicated, no policy, no control mechanism…’* *‘And after earthquake those focal point started being created which led the whole mental health system in Nepal, like it gave direction to everything.’* *‘Now PHCRD is taking leading role and we brought all NGOs and INGOs in one umbrella and then we are working now with coordination side by side.’* *‘Our [mental health] policy was not revised for more than a decade. In my view the earthquake showed us the feeling, the need to address that. Now that policy [revision] has moved forward a little… If the WHO did not coordinate here, the policy would have not have reached this point, but the earthquake is the reason WHO came [to have a stronger presence] here.’*
Financing	• Government funding for mental health mainly limited to one state psychiatric institution	• Government allocated a separate budget for mental health for the first time, which will cover implementation of the Community Mental Health Care Package in seven additional districts • Increased funding from NGOs, international NGOs (INGOs) and international donors • Increased funding from other sectors and intersectoral collaboration; e.g., the Department of Women, Children and Social Welfare integrated counselling into one-stop crisis management centres for women experiencing violence	*‘…for the first time government took some mental health program in their red book [annual budget planning] program, for the first time government gave budget.’* *‘Earthquake can be catalyst…. For example, in Canada someone wanted to give us money…but they could not raise [funds for] mental health. But after earthquake, people gave a lot of money.’* *‘Bringing people [with mental illness] to Nepalgung or Kathmandu [for treatment] is quite expensive…but post*-*earthquake when mental health system is so much discussed and talked about, and many actors, also even protection clusters, they started assigning certain budgets for issuing and bringing chronic cases in the residential treatment.’*
Human resources	• Limited training on mental health in government health education system • Psychosocial workers and counsellors trained mainly by NGOs	• Primary health care workers in public system in 14 affected districts trained using mhGAP • Female Community Health Volunteers and other frontline workers trained on identification and referral • New psychosocial counsellors and community psychosocial workers trained • Many trained on offering psychological first aid • Efforts underway to engage medical schools in strengthening mental health curriculum of medical students^a^	*‘One important thing was introducing mhGAP in government in Nepal.* ^b^ *Though it has been used in different NGOs in Nepal but government ha[d]n’t acknowledged it and ha[d]n’t started training for that. WHO coordinated with government and we started training the medical officers that were appointed in those [affected] areas.’*
Psychotropic drugs	• 6^c^ psychotropic drugs on the government list of freely available essential drugs	• 12^c^ psychotropic drugs on free drugs list to be provided free of cost in districts where service providers have been trained on assessment and management of mental disorders	*‘…if earthquake has not been there, forget about these changes. Even that 5* ^c^ *medicines also would have been eliminated or not given priority.’*
Information and research	• PFA and Inter-Agency Standing Committee (IASC) guidelines had been translated in Nepali • Some relevant experience/protocols from flood-related interventions • Some intervention effectiveness research • Community Informant Detection Tool (CIDT) had been developed for some disorders and validated in Nepali^a^	• Consolidation of information/research available before the earthquakes • Needs assessments and other research conducted, including on PTSD prevalence • A version of CIDT for general distress was developed and disseminated • First national mental health epidemiological survey is being planned, led by the Nepal Health Research Council of MOH • Information, education and communication materials on mental health prepared and widely disseminated in public • Desk review of information relevant to MHPSS intervention in Nepal prepared^a^	*‘We had already IASC guidelines, we had PFA and psychological factors already in place, we have other researches that has been published, many NGOs had published their own materials for orientation of mental health and psychosocial awareness…. Those things were not seen before the earthquake but after earthquake it was seen in system.’* *‘What earthquake did was, it gathered one to act together so the information was gathered. As a psychiatrist…I didn’t know [PFA] was translated…. WHO gathered everyone as subcluster and everyone came to know things that have gained in the mental health field in Nepal.*’
Service delivery	• Community mental health model/training curricula existed • Service delivery NGO-led and not integrated into government system • Services concentrated in cities • Selected modules of mhGAP Intervention Guide had been adapted to the Nepal context^a^	• Translation and adaptation of mhGAP version 2 under PHCRD and design of training manual for medical officers and health assistants under National Health Training Centre^a^ • Revised Standard Treatment Protocol for delivery of mental health services in primary care settings based on mhGAP and international guidelines endorsed by PHCRD • Primary care providers in 14 affected districts trained in assessment, management, and follow-up for mental health problems • Frontline workers trained in identification and referral • Increased public awareness led to increased presentation at services • Mass conversion disorder intervention manual developed to facilitate intersectoral action from health and education sectors	*‘[Before the earthquake] we couldn’t even cover each and every district as single resources couldn’t be sent to each and every district. But later good thing was with partnership with government, INGOs and NGOs, other organizations, and we started sending resources to various districts…. More than clinical support, psychosocial support was the best, it was all over the earthquake affected areas.’* *‘Before, many people wouldn’t address [mental] illness. After the earthquake people who hadn’t been to a psychiatrist, mental health professional, finally started to come, the earthquake also boosted that awareness.’* *‘Now after the earthquake many reports of those [mental health cases] came. Wherever mental health actors are in the community, there case reports have increased.’*

^a^These developments were not explicitly mentioned in focus group but were added subsequently by authors of this article who were present at the focus group and directly contributed to these initiatives

^b^mhGAP had been integrated into the government system in one district (Chitwan) prior to the earthquakes [26–28]

^c^This list includes two forms of the same drug, Diazepam (injection and tablet), so some stakeholders considered the total number of psychotropic drugs on this list to be 5 and 11 before and after the earthquake, respectively

#### Sustainability of post-earthquake achievements

Participants expressed a range of viewpoints related to the sustainability of system reform progress in the post-earthquake context. Some participants were optimistic, citing increased awareness of the importance of MHPSS agendas:

I think sustainability doesn’t just mean some steps only, it also means awareness. Awareness never fades…. The awareness that everyone has developed – not just in the general population, it also happened in policy makers – for this reason I am seeing that mental health programs have some sustainability.

Several others noted the important convening role that the WHO mental health focal point can play in ensuring sustainability of post-earthquake achievements. This focal point noted:

My role is to convene such meetings and whatever interesting project happens– [consider] how can we mainstream this, how can we bring this into the light, how can we attempt to take this up, focusing on sustainability issues. On the behalf of WHO, I have been working towards such activities. In hopes that the national system will take up lessons learned and those things that are feasible, WHO is attempting to play a convening role and act as a technical advisor to the Ministry of Health.

Despite these important gains, many raised concerns over the short-lived nature of post-earthquake funding for MHPSS. In the words of one participant, ‘In the beginning many were addressing [mental health]. But after about 1 year funds ran out, the number of NGOs, INGOs became few’. All stakeholders agreed that working with the government and within the existing health system was essential to ensure program sustainability. One NGO representative described making plans for a transition of responsibilities to the government from the outset of post-earthquake projects, anticipating that disaster related funding would soon run out:

When we initiated post-earthquake programs…we thought we need to work together with Ministry of Health and district health system, so that they would be later on accountable to mainstream…mental health in their system, so the programs that we had initiated together with ministry of health… also included district health officers in our program.

However, actually achieving this transition sometimes proved difficult. One participant described how poor coordination between governmental and non-governmental stakeholders led to the loss of patients from services:

In my experience in [NGO]’s program in [district name], there were 100 listed patients. When the [NGO]’s programs were phased out, while we were taking over the program there were only two to three people in our OPD. Without collaborating with the government the patients do not show up later. I had noted and kept with me the names registered at that time and those patients have still not come to see us. Therefore good coordination is required.

In some cases, the government lacked resources to fill in where NGOs left off, particularly with regard to supervision by mental health specialists: ‘…there is an issue of how to do the supervision and monitoring part that [NGO] had been doing’; ‘[Un]til now there are very few psychiatrists in the government, you cannot see psychiatrists in the regional level. That impacted a lot into particularly maintaining the referral mechanisms’. It was further noted that specialized human resources remain concentrated in Kathmandu or other big cities and that issues with the availability of medications persisted.

Concerns were also raised about government ownership of projects, particularly the integration of mental health in primary care. One participant pointed out:

Prescriber, non-prescriber…both have been trained. But where there is a gap seen is that this is a program brought from outside, it is not from the government system. If it had come from the government system, it would have fallen within their [health workers’] duty. It hasn’t happened yet that our health workers perceive that mental health, psychosocial support is within their job description.

Without full government ownership, participants stressed, health workers were unlikely to continue to provide adequate care once initial supports for trainees were phased out:

During supervision they do a little, after that [health workers] have a custom of setting the [mentally] ill aside, saying ‘When are you [specialists] coming? We’ll call them here for you’. They take no ownership.

For the government to take ownership in the long term, it should define this as a part of [health workers’] duty in their job description. The external actors like us and WHO have been stressing this to the government. [Currently] it depends on [the trained health workers’] willingness. If the health workers are motivated, they are giving service. If they are not motivated– they came, training was given, the program was phased out, and it’s all over.

This problem was seen to be compounded by the fact that trained primary care workers are frequently transferred to other districts where supervision and referral mechanisms are lacking.

Finally, participants stressed the need to increase the budget allocated by the government for mental health. One stakeholder noted,

The governmental budget of two *karod* [twenty (rounded from 18) million Nepali rupees] has been allocated, now if five *karod*, 10 *karod* are allocated from next year it will be easy for government takeover to be sustainable. NGOs are donor driven, therefore we can’t know when it will collapse. So it will be sustainable long-term if the government takes over while increasing the budget step-wise….

In addition to sustaining existing programs, participants noted that additional funding will be needed to scale up services to the national level. The current budget proposal will cover a comprehensive care package in only seven of Nepal’s 77 districts and psychotropic medications on the free drugs list will only be available for free in districts where prescribers have received mental health training based on mhGAP.

## Discussion

This study drew on documentation of post-earthquake MHPSS response activities and outcomes as well as the perspectives of key stakeholders to shed light on the extent to which ‘building back better’ has been achieved in Nepal. Available outcome data, though limited, supported claims of governmental and non-governmental actors in the mental health sector that the disaster served as a platform for accelerating processes of national mental health system building. With regard to direct effects, documentary evidence reflected long-term contributions in the area of human resources and service delivery. The number of primary care providers trained on mental health treatment more than doubled through response activities of the three most active organizations alone [17]. The number of community psychosocial workers also increased substantially while the low number of new psychosocial counsellors trained, in light of the large numbers of beneficiaries of psychosocial counselling and support, suggests NGOs heeded calls to mobilize an existing cadre of psychosocial counsellors built up during previous emergencies [38]. Tens of thousands were trained on providing psychological first aid, representing a substantial contribution towards preparedness for future disasters [14]. Psychoeducation activities together with the establishment of new identification and referral mechanisms at the community level were credited with increasing help-seeking for mental health problems.

In addition to direct outcomes of response activities, stakeholders attributed a number of important developments during the recovery stage and beyond to increased attention to MHPSS issues occasioned by the disaster. Participants described the appointment of focal points for mental health in the government and the WHO Country Office as particularly crucial in facilitating prioritisation and improved coordination of MHPSS activities. The government has now allocated a budget for district-level mental health care for the first time in history; added six psychotropic drugs to the free drugs list; and prepared a revised national mental health policy, a standardized treatment protocol, and a community mental health care package. The mental health focal point in the WHO Country Office has supported these steps and plays an essential coordinating role among diverse stakeholders. Non-health sector governmental and non-governmental organizations have also increasingly supported MHPSS services.

Participants noted that many of these developments were made possible by the groundwork laid by mental health NGOs in Nepal prior to the earthquake. In the years before the disaster, mental health NGOs had made important strides towards elaborating a collective vision for a national community mental health system and had been actively providing services, developing resources, and advocating for policy and budgeting revisions. Overall, findings of this study confirmed Sherchan and colleagues’ observation that, ‘The heightened attention of policy-makers and health administrators, owing to the “tipping point” of the earthquake, has been utilized strategically to ensure actions on interventions that had already been advocated for a considerable time but were in limbo’ [36]. This emerging consensus calls into question Jayawickrama and Rose’s recent assertion that Nepal’s post-earthquake MHPSS response was guided by ‘outside mental health experts’ with limited understanding of local cultural and social context [39]. Their critique appears to overlook the central role of experienced Nepali NGOs, clinicians, and service user advocates in the post-disaster response as well as these stakeholders’ important contributions to setting and advancing the national mental health agenda over the past decade [18, 36].

Considering guidelines and evidence on transitioning from emergency response to sustainable development, it appears that a number of best practices were observed in Nepal. NGOs involved the government and planned for sustainability at an early stage by striving to integrate mental health into primary care [13, 14]. Strengthening community self-help and social support was achieved through the widespread training of lay people, community leaders, and frontline workers on basic psychosocial support [14]. Attention was given to updating mental health policy and legislation [13, 15]. Multiple points of intervention from the community to the clinic were adopted, including non-health sector programs targeting vulnerable populations. Finally, existing knowledge, capacity, and resources (e.g., psychosocial counsellors, female community health volunteers) were leveraged [15].

Yet our analysis also highlights remaining challenges in Nepal’s effort to build back better. Integration of MHPSS care into the national system has only been achieved in 15 (14 post-earthquake) of Nepal’s 77 districts and the budget now allocated by the MOH guarantees coverage of only seven additional districts. Without national scale up, trained primary care workers who are transferred to other districts may be unable to continue treating mental health problems due to lack of supervision, referral mechanisms, and psychotropic drugs. In districts where mental health services have been integrated, MHPSS has not yet been included in the job descriptions of health workers. Moreover, no response activity trained new mental health specialists (psychiatrists or psychologists), leading to gaps in supervision, monitoring, and referral mechanisms. Available guidelines suggest building sustainable capacity requires not only providing, but institutionalizing trainings [13], and that ongoing supervision and professional development opportunities for those who received training are essential [40]. Finally, it is noteworthy that no provisions have been made for integrating the many psychosocial care providers mobilized in the response into the primary care system. This is inconsistent with guidelines suggesting that the optimal mix of services in mental health systems should foreground non-specialized community psychosocial support [10]. A narrow focus on primary care, without strengthening other tiers of the MHPSS system, creates a risk that mental health care in these settings will become highly medicalized [40].

Taken together, these findings resonate with Patel and colleagues’ caution that government buy-in is not always sufficient to ensure an effective transition from emergency response to sustainable MHPSS system development; steps must be taken to strengthen capacity within the government, sustain political will over time, and cultivate functional relationships among stakeholders with divergent interests [15]. The WHO Country Office can play an important role in bringing these stakeholders to the table and transforming experience into guidance for the way forward. Looking ahead, the WHO Country Office has made plans to support the MOH in developing a basic health care package for Primary Health Centres nationwide which will incorporate mental health services, as well as to build the capacity of provincial mental health services to respond to disasters by establishing networks of hub and satellite hospitals. As they have historically, NGOs in Nepal can continue to lobby the government for sustainable mental health system development and to offer their expertise and experience to this end [18]. Increased awareness of MHPSS issues at all levels of society offers grounds for optimism that building a sustainable community mental health care system in Nepal is achievable.

### Limitations

Given the aims and timing of this study, we did not adopt a systematic literature review methodology. The grey literature review portion of this study relied on data that were voluntarily reported by a limited number of institutions; we moreover only included grey literature from sources which were explicitly concerned with sharing information on the MHPSS response. Findings may not accurately or completely reflect response activities and stakeholders and it is possible that activity categories such as ‘psychosocial support’ were not used in a consistent way across reporting organizations. The poor quality of documentation may also have affected determinations of which organizations were most active in the response. Qualitative data represent the perspectives of a limited number of stakeholders who accepted our invitation to participate in a focus group. While the authors’ extensive first-hand knowledge of Nepal’s mental health sector affirms the central role of the organizations who contributed both quantitative outcome data and qualitative data, representation of a wider range of organizations would have allowed a more robust analysis.

## Conclusion

Building back better has been achieved to varying extents in different districts and at different levels of the mental health system. Major achievements include training of primary care providers and psychosocial workers in affected districts; appointment of mental health focal points in the government and WHO Country Office; the addition of six psychotropic drugs to the free drugs list; and the revision of mental health plans, policy, and financing mechanisms. Concerns remain that government ownership and financing are insufficient to sustain services in affected districts and scale them up to non-affected districts. NGOs and the WHO Country Office must continue to closely support the government to ensure that post-disaster developments maximally contribute to realising the vision of a sustainable community mental health system in Nepal.

## Abbreviations

5W: ‘Who, What, When, Where, for Whom’ forms documenting post-earthquake response activities and submitted to disaster response cluster or subcluster chairs
CIDT: Community Informant Detection Tool
IASC: Inter-Agency Standing Committee
INGO: International non-governmental organization
mhGAP (IG/HIG): Mental Health Gap Action Program (Intervention Guide/Humanitarian Intervention Guide)
MOH: Ministry of Health
NCD: non-communicable disease
NGO: non-governmental organization
PFA: psychological first aid
PHCRD: Primary Health Care Revitalisation Division
WHO: World Health Organization
